# Evaluation of health equity frameworks in telehealth and digital health: a systematic review and narrative synthesis

**DOI:** 10.3389/fpubh.2025.1690117

**Published:** 2026-01-06

**Authors:** Siyu Wang, Anagha Killedar, Amy Von Huben, Sarah Norris, Andrew Wilson

**Affiliations:** Faculty of Medicine and Health, Sydney School of Public Health, Leeder Centre for Health Policy, Economics and Data, The University of Sydney, Sydney, NSW, Australia

**Keywords:** determinants of health, digital health, health equity, healthcare disparities, health equity frameworks, telehealth, telemedicine

## Abstract

**Introduction:**

The rise of telehealth and digital health solutions has been promoted as a transformative approach to bridging healthcare access gaps. However, these technologies may introduce new disparities related to digital literacy, Internet access, and technology affordability. While several health equity frameworks exist for traditional healthcare delivery, their applicability to telehealth contexts remains unclear.

**Methods:**

We systematically searched MEDLINE, CINAHL, and Scopus databases for peer-reviewed articles discussing health equity frameworks relevant to telehealth and digital health. A narrative synthesis approach was employed, complemented by a tailored quality appraisal assessing Clarity, Theoretical Basis, Comprehensiveness, and Applicability to telehealth. Framework elements were categorized into drivers, measures, and enablers/modifiers of health equity.

**Results:**

The search yielded 707 records; after removing 25 duplicates, 27 full-text articles were assessed, and 17 were included in the final synthesis. Thirteen unique health equity frameworks were identified. Six frameworks were explicitly developed for digital health, telehealth, or eHealth contexts, while seven were general health equity frameworks with potential applicability to telehealth. Eight frameworks demonstrated moderate-to-high quality scores. Across frameworks, notable gaps remain in addressing telehealth-specific challenges and in providing actionable guidance for real-world implementation.

**Conclusion:**

Existing frameworks provide valuable insights but require adaptation to address telehealth-specific challenges fully. Tailored frameworks that incorporate digital determinants of health, prioritize equitable access to technology, and facilitate system integration are essential. These advancements will enable telehealth to effectively reduce health disparities and advance equity in healthcare access.

**Systematic review registration:**

https://www.crd.york.ac.uk/PROSPERO/view/607575, Identifier PROSPERO (CRD42024607575).

## 1 Introduction

### 1.1 Background

Systematic differences in health by socioeconomic or cultural characteristics, henceforth referred to as health inequity, remain a significant concern worldwide. Health inequities are largely determined by socioeconomic, geographic, and demographic factors, but these can, in part, act through differences in the quality and accessibility of healthcare services for different population groups (1). In recent years, the rise of digital health solutions, particularly telehealth, has been heralded as a transformative approach to bridging these healthcare access gaps (2). Telehealth, defined as the use of digital communication technologies to deliver health services remotely, offers the potential to extend healthcare access to underserved and remote populations, thereby addressing some of the inequities in traditional healthcare delivery (3).

However, the introduction of telehealth also presents unique challenges that must be addressed to ensure that its use contributes positively to health equity. While telehealth can mitigate geographic barriers and improve access to specialist care, it can also introduce new disparities related to digital literacy, Internet access, and the affordability of technology (4). These challenges highlight the importance of applying a structured equity framework to telehealth to ensure that digital health innovations do not exacerbate existing inequities.

Several health equity frameworks have been developed to assess equity considerations in healthcare delivery more broadly. However, relatively few of these frameworks are explicitly designed for telehealth or digital health contexts. This raises the question of whether existing frameworks apply to telehealth and, if not, whether modifications or new frameworks are needed to evaluate digital health equity adequately. This study systematically reviews existing health equity frameworks, including those not originally developed for telehealth, to assess their relevance and applicability to telehealth contexts. By analyzing these frameworks, we identify whether telehealth introduces unique equity considerations that require adaptations to existing frameworks.

This study will deepen understanding of the concepts of health equity in relation to emerging digital technologies and provide practical insights for policymakers, healthcare providers, and researchers to ensure that equity considerations are incorporated into the implementation of telehealth. By continually refining telehealth practices and policies from an equity perspective, we can move closer to a healthcare system that reduces disparities rather than widens them.

### 1.2 Objectives

The objectives of this systematic review are:

(1) Critical Evaluation of Existing Frameworks: Identify and analyze current frameworks that assess equity in healthcare access, identify common factors and differences, and evaluate the applicability of these frameworks to telehealth.
(2) Clarify Telehealth-Specific Equity Considerations: Synthesize observations across the included frameworks to identify any additional dimensions or considerations specific to telehealth that may be absent from general health equity frameworks.

### 1.3 Definitions

To ensure clarity, this review uses the following definitions:

Telehealth: Telehealth includes healthcare services provided using audio and video technology and is a subset of e-health (5). Telehealth typically involves direct patient–provider interactions (e.g., video consultations, remote monitoring) and is focused on the clinical delivery of care (6).
Digital Health: Digital health refers to the use of information and communication technologies in medicine and other health professions to manage disease and health risks and promote health, and it is broad in scope, including the use of wearable devices, mobile health, telemedicine, and health information technology and telemedicine (7).
Technology (in the context of healthcare): Refers to the digital tools, platforms, and infrastructures used to support healthcare delivery (8).
Health Equity Framework: A structured theoretical model or conceptual approach that identifies, analyzes, and addresses the factors contributing to unfair health disparities across population groups. These frameworks provide a lens to examine how social, economic, cultural, and technological determinants interact to shape equitable access to health services and outcomes. They typically include three components: (1) key drivers or determinants of health equity, (2) measures or indicators for assessing equity, and (3) enablers or interventions that can modify equity outcomes.

## 2 Methods

This systematic review follows the Preferred Reporting Items for Systematic Review and Meta-Analysis (PRISMA) statement (9). This review was prospectively registered with the International Prospective Register of Systematic Reviews (PROSPERO). A narrative synthesis of findings has been undertaken according to the methods of Popay et al. (10).

### 2.1 Eligibility criteria

To ensure the inclusion of relevant and high-quality literature, we have established specific inclusion and exclusion criteria (see Table 1). We included both telehealth-specific and general health equity frameworks for several methodological reasons. First, the limited number of frameworks explicitly developed for telehealth/digital health contexts necessitated a broader scope to ensure comprehensive coverage of relevant theoretical constructs. Second, general health equity frameworks provide foundational insights into core determinants of health disparities that remain relevant in digital health contexts, such as socioeconomic status, geographic location, and cultural factors. Third, examining general frameworks allows us to identify which elements translate effectively to telehealth settings and which require adaptation, providing crucial insights for framework development. Finally, this inclusive approach enables us to assess the theoretical maturity of health equity concepts and identify specific gaps that future telehealth-specific frameworks must address.

**Table 1:** Inclusion and exclusion criteria for peer-reviewed journal articles.

Criteria category	Criteria	Description
Inclusion criteria	Content	Articles must explicitly discuss, evaluate, or apply health equity frameworks, including those not originally designed for telehealth/digital health, but with potential relevance to digital health equity.
	Language	Only literature written in English will be included to ensure accurate comprehension and analysis.
	Types of literature	Peer-reviewed academic articles published in scholarly journals.
Exclusion criteria	Non-academic literature	Excludes non-academic or non-peer-reviewed literature such as news articles, blog posts, opinion pieces, editorials, and gray literature (e.g., reports and dissertations).
	No health equity framework	Excludes literature that does not discuss, evaluate, or apply health equity frameworks or models, even if it involves telehealth or digital health issues.
	Lack of health equity discussion	Studies focus solely on clinical outcomes, technological aspects, or healthcare access without addressing equity considerations.
	Language	Non-English articles.

Operational definitions: In this review, we use telehealth as an umbrella term for the remote delivery of health-related services using information and communication technologies, including clinical care and non-clinical services. We use telemedicine to refer specifically to remote clinical services (e.g., diagnosis, treatment, and follow-up). We use digital health to refer broadly to the use of digital technologies to support health and healthcare, which may include telehealth/telemedicine as a subset. For consistency, the term “telehealth” is used throughout when referring to the review’s primary context unless a framework uses alternative terminology.

### 2.2 Information sources and search strategy

A comprehensive literature search was conducted in MEDLINE (Ovid), CINAHL (EBSCOhost), and Scopus to identify peer-reviewed articles on health equity frameworks in telehealth/digital health (Additional File 1). The initial search was run in July 2024 and updated in November 2024 (exact run dates and database yields are reported in Additional File 1).

The search strategy was developed with the assistance of a medical librarian and included a combination of Medical Subject Headings terms and keywords related to health equity frameworks and telehealth. The search terms were adjusted for each database to account for differences in indexing and search functionalities (see Additional File 2). No date or geographical restrictions were applied to maximize the retrieval of relevant literature. Only articles published in English were considered to ensure accurate comprehension and analysis.

In addition to electronic database searches, we manually searched the reference lists of all included articles and relevant reviews to identify additional studies that may not have been captured in the database searches. Gray literature and non-peer-reviewed sources were excluded as per the inclusion criteria.

### 2.3 Selection process

**Figure 1:**
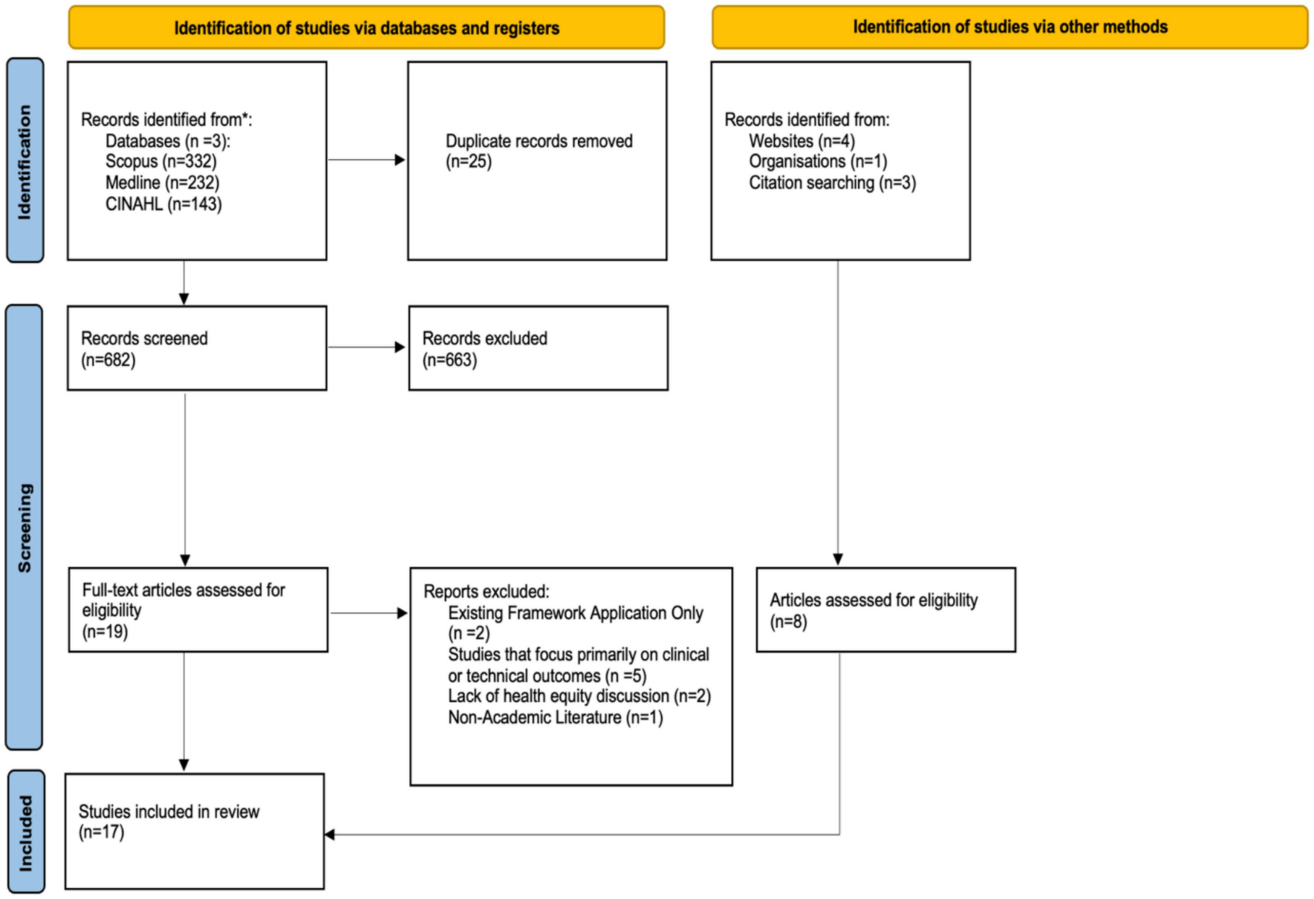
PRISMA flow diagram of study selection. Database searches were run on 2 July 2024 and re-run on 11 November 2024; records from both runs were combined prior to de-duplication and screening.

The study selection process is illustrated in a PRISMA flow diagram (Figure 1). All records retrieved from the database searches were imported into EndNote X9, and duplicates were removed. The study selection process was conducted in two stages:

Stage 1: Title and Abstract Screening.

Two reviewers (SW and SN) independently screened the titles and abstracts of all retrieved records against the inclusion and exclusion criteria outlined in Table 1. Studies that clearly met the inclusion criteria or where eligibility was uncertain were advanced to the full-text review stage. Discrepancies were resolved through discussion by a third reviewer (AW).

Stage 2: Full-Text Review.

The same two reviewers independently assessed the full-text articles for eligibility. Reasons for exclusion at this stage were documented in detail. A full-text exclusion log (*n* = 10), including citations and reasons for exclusion, is provided in Additional File 2 (Part B). Disagreements were resolved through discussion, and when consensus could not be reached, a third reviewer (AW) was consulted to make the final decision.

### 2.4 Data extraction

In our review, the data extraction process was designed to accurately summarize and assess key information on health equity frameworks from the selected literature. Data extraction was conducted using a piloted extraction form. One reviewer (SW) extracted data from all included articles to ensure consistency. To minimize extraction or transcription errors, extracted information was cross-checked against the source articles during synthesis and table preparation, and uncertainties were discussed within the author team. For each selected article, we extracted the following information:

1) Name of the health equity framework or model: the specific title or designation of the framework discussed in the article.
2) Primary purpose of the framework: the main objectives or intended outcomes that the framework aims to achieve in the context of health equity.
3) Key elements of the framework: the essential components, constructs, or domains that constitute the framework.
4) Theory underpinning the framework: the theoretical foundations or conceptual models that inform the development of the framework.

Additionally, we categorized the factors covered within each framework into three groups, derived from established health equity literature and empirical research: (1) drivers of health equity, which are core factors that lead to disparities, such as socioeconomic status (SES), geographical location, and cultural background (11, 12); (2) measures, which are specific indicators or methods used to assess health equity, like inequalities in health outcomes and accessibility of services (13, 14); and (3) enablers/modifiers of health equity, such as policy interventions, technological innovations, and educational initiatives (15). They represent factors or interventions that can directly affect health equity, either by strengthening or hindering it.

### 2.5 Quality assessment

As the review focuses on frameworks rather than empirical research studies, traditional risk-of-bias assessment tools were not appropriate. Instead, we developed a tailored quality assessment approach to evaluate the Clarity, Theoretical Basis, Comprehensiveness, and practical Applicability of each framework to telehealth. The tool included explicit decision rules (score anchors) for each criterion (0–3) to support consistent scoring of conceptual frameworks. The complete scoring rubric and a blank scoring template are provided in Additional File 4.

#### 2.5.1 Criteria

We assessed the quality and applicability of the frameworks using specific criteria:

1) Clarity of purpose: whether the framework’s objectives are clearly defined.
2) Theoretical basis: the extent to which the framework is grounded in existing theory and evidence.
3) Comprehensiveness: coverage of relevant factors influencing health equity (from our previous research) (16).
4) Applicability: the framework’s potential for practical application in telehealth settings.

#### 2.5.2 Scoring system

Each criterion was scored on a 4-point scale:

0 (not met): The criterion is not addressed: Red.
1 (partially met): The criterion is addressed, but with significant limitations: Orange.
2 (mostly met): The criterion is adequately addressed with minor limitations: Light green.
3 (fully met): The criterion is fully addressed without significant limitations: Dark green.

#### 2.5.3 Overall quality classification

The scores for all four criteria were then summed to produce a total quality score ranging from 0 to 12. We classified each framework into one of four quality categories based on the total score:

High quality (10–12): Dark green.
Moderate quality (7–9): Light green.
Fair quality (4–6): Orange.
Low quality (0–3): Red.

#### 2.5.4 Assessment process

Three reviewers (SW, AW, and AK) conducted a quality and applicability assessment for all frameworks. SW independently evaluated all frameworks, while AW and AK each assessed a subset (AW evaluated nine frameworks and AK evaluated eight frameworks). Any differences in assessments were discussed among the reviewers, and if agreement could not be reached, a fourth reviewer (SN) made the final judgement.

### 2.6 Data synthesis

To assess the relevance of general health equity frameworks to telehealth or digital health, we conducted a sub-analysis distinguishing between:

1) Frameworks explicitly designed for telehealth or digital health.
2) General health equity frameworks with potential applicability to telehealth or digital health.

We synthesized the findings narratively, focusing on analyzing and comparing the similarities and differences among the identified health equity frameworks. The narrative synthesis was structured around the objectives of the review and followed the approach outlined by Popay et al. (10). The data synthesis was conducted as follows:

Step 1: Developing a preliminary synthesis.

We developed a preliminary synthesis by organizing data from the included studies into comprehensive tables that summarized the characteristics and key components of each framework.

Step 2: Common factors and key differences.

We compared the frameworks to identify similarities and differences in how they address health equity factors.

Step 3: Thematic analysis of framework components.

The factors within each framework were categorized into the three groups identified during data extraction: drivers of health equity, measures, and enablers/modifiers of health equity (as mentioned previously).

Step 4: Applicability of frameworks to telehealth settings.

To evaluate the relevance of the frameworks, we examined their applicability to telehealth-specific equity challenges.

## 3 Results

### 3.1 Study selection

Figure 1 shows the article selection process for this review. The initial database search yielded 707 articles. After removing 25 duplicates, 682 articles remained. Screening titles and abstracts excluded 663, leaving 19 for full-text review. Supplementary searches added 8 more articles, bringing the total to 27 for full-text assessment. Of these, 17 met the inclusion criteria and were included in the final analysis.

### 3.2 Characteristics of included studies

We identified 13 unique health equity–related frameworks across the 17 included articles. Of these, six frameworks were explicitly developed for digital health, telehealth, or eHealth contexts (Table 2). At the same time, the remaining seven were general health equity frameworks originally developed for non-digital healthcare settings but considered potentially applicable to telehealth and digital health. Additional File 3 provides a detailed summary of all 13 frameworks.

**Table 2:** Strengths and limitations of digital health-specific equity frameworks (*n* = 6).

Framework	Key strengths	Key limitations
Kepper et al. (23) A Model for Advancing Digital Health Access to Foster Health Equity	Comprehensive access dimensions, user-centered design, and focus on sustainability.	Limited telehealth-specific focus on operational challenges.
Crawford and Serhal (18) Digital Health Equity Framework	Addresses digital determinants of health, integrates equity into health systems, and ensures person-centered care.	Lack of implementation metrics for telehealth.
Groom et al. (33) Digital Health Equity-focused Implementation Research Model	Focus on planning, design, implementation, and equity-focused outcomes.	High resource demands for equity assessment.
Antonio and Petrovskaya (32) eHealth Equity Framework	Contextual and systemic focus, life course perspective, intermediary determinants of health.	Limited focus on telehealth service integration.
Richardson et al. (27) Framework for Digital Health Equity	A multi-level approach that addresses individual-to-societal factors and considers algorithmic bias.	Minimal guidance on practical implementation for telehealth.
Foley et al. (26) Suggested Pathways of Access, Use, and Benefit from Digital Health Services	Clear pathway (access → use → benefit), trust and literacy focus, and hybrid care integration.	Overemphasis on population-level services, lacking nuance in individual telehealth interactions.

### 3.3 Quality assessment results

Additional File 4 includes the appraisal rubric and scoring template, as well as the quality assessment results for all identified frameworks. Each framework was assessed against four criteria—Clarity of Purpose, Theoretical Basis, Comprehensiveness, and Applicability—scored on a scale from 0 (not met) to 3 (fully met). The total possible score ranged from 0 to 12, with higher scores indicating higher overall quality.

Overall, the majority of the frameworks demonstrated moderate to high quality. Eight frameworks achieved a total score of 10 or above, indicating strong Theoretical Grounding, Clarity of Purpose, and Applicability to health equity assessments. Four frameworks were given the highest score of 11, reflecting robust theoretical foundations and applicability to both general healthcare equity and digital health contexts. However, Comprehensiveness emerged as the weakest criterion across many frameworks, with several receiving low scores in this domain. Specifically, the five frameworks scored only 1 for Comprehensiveness, indicating that they lack holistic consideration of key dimensions of health equity or fail to integrate the broad determinants related to digital health equity. Among the lower-scoring frameworks, O’Neill et al. (17) (PROGnosis RESearch Strategy [PROGRESS] framework) scored the lowest overall (6 points), mainly due to its limited Comprehensiveness and weak theoretical foundation. The PROGRESS framework focuses narrowly on social stratifies (e.g., place of residence, race, gender, socioeconomic status) but does not fully integrate other systemic or structural factors influencing digital health equity, such as healthcare system barriers, digital infrastructure, or policy interventions. Similarly, Crawford and Serhal (18) (The Digital Health Equity Framework) received a total score of 7, with particularly low scores in Clarity of Purpose and Comprehensiveness. While the framework explicitly addresses digital health, its scope is limited to broad structural considerations. It does not offer detailed, actionable guidance on how to measure or operationalize equity outcomes in telehealth settings.

Other frameworks that scored moderately in Comprehensiveness (score of 1–2) include Dover and Belon (29) (Health Equity Measurement Framework) and the World Health Organization (WHO) (24) (Conceptual Framework for Action on the Social Determinants of Health). While these frameworks provide strong theoretical models for analyzing general health equity, they fall short in addressing digital determinants of health, access to telehealth services, and technology-specific barriers. This suggests that general health equity frameworks, while foundational, may require adaptation to assess telehealth-specific equity considerations adequately. In contrast, the highest-scoring frameworks [e.g., (19–21)] incorporated more comprehensive multi-level assessments that integrated individual, community, and systemic factors.

## 4 Narrative synthesis

### 4.1 Analysis of different frameworks

#### 4.1.1 Common factors and key differences

Additional File 5 highlights the common factors and differences found across the frameworks. We consider a factor common if it appears in six or more frameworks; otherwise, it is classified as a difference.

##### 4.1.1.1 Common factors

All frameworks acknowledge that health outcomes are not solely determined by medical care but are also shaped by social, economic, and environmental factors. Seven of the 13 frameworks emphasize various dimensions of access to healthcare services (18–20, 22–26). Cultural and linguistic considerations appear in 11 frameworks (17, 18, 20, 21, 23–33). Six frameworks underscore the importance of policies and systemic reforms (18, 20–22, 24, 32). Among frameworks focused on digital health or eHealth, digital determinants of health are consistently recognized as equity drivers (18, 23, 26, 27, 32, 33). Additionally, six frameworks incorporate feedback loops, continuous evaluation, and adaptation to facilitate sustained impact on health equity (18, 20, 22, 23, 32, 33).

##### 4.1.1.2 Key differences

Only one digitally focused framework explicitly mentions algorithmic bias, data standards, and hidden technology biases (27). Three frameworks stress the importance of patient engagement and trust (18, 23, 33). Frameworks vary in how they address measurement and evaluation. More traditional frameworks (e.g., Aday and Andersen model) center on healthcare utilization metrics, while recent digital health frameworks stress the importance of collecting and analyzing digital equity data, such as usage patterns, technology adoption rates, and culturally tailored evaluation metrics (18, 23, 33). Some frameworks remain broad and adaptable across multiple contexts (e.g., PROGRESS-Plus, which includes place of residence, race/ethnicity, occupation, gender, religion, education, socioeconomic status, social capital, plus additional context-specific factors), enabling their use in numerous settings and populations. Others are highly specialized, focusing on integrating equity considerations throughout the lifecycle of specific health information technologies (e.g., eHealth Equity Framework).

##### 4.1.1.3 Thematic analysis of framework components

We categorized elements from all frameworks into Drivers, Measures, and Enablers/Modifiers (this classification is based on prior definitions). Table 3 summarizes typical elements from various frameworks and categorizes them accordingly.

**Table 3:** Categorization of framework components across included frameworks (*n* = 13): drivers, measures, and enablers/modifiers.

Category	Elements (from various frameworks)
Drivers (core factors)	- Socioeconomic status (income, education, and occupation) - Geographic location (urban–rural differences and regional characteristics) - Race/ethnicity, cultural background, and language differences - Social stratification and structural inequalities - **Digital literacy, technology access, and affordability of devices**
Measures (indicators)	- Healthcare utilization rates (e.g., consultation rates and use of preventive services) - Indicators of disparities in health outcomes (morbidity, mortality, and disease burden across different groups) - **Digital health service usage indicators (e.g., rates of electronic health record usage, telehealth visits, adoption rates)** - Patient satisfaction, trust, and cultural alignment measures - Quantitative measures of health inequality
Enablers/modifiers (promoters/changers)	- Policy interventions and systemic reforms (policy recommendations, legislative action, and funding aimed at reducing inequalities) - **Technological innovations and design improvements (user-centered design, accessibility enhancements, training, and technical support)** - Community outreach and communication strategies (raising awareness and reducing information gaps) - **Educational programs to enhance digital literacy** - Interdisciplinary collaboration and stakeholder engagement

Elements relevant specifically to digital health contexts are in bold.

##### 4.1.1.4 Drivers

All frameworks identified fundamental social determinants as core drivers of health equity. These included socioeconomic status, race/ethnicity, geographic location, language, cultural norms, and structural inequalities within health systems. Frameworks adapted for digital health further recognized digital literacy, access to broadband, and the affordability of devices as critical new drivers of equity in the digital health context (18, 23, 26, 27, 32, 33).

##### 4.1.1.5 Measures

Common measures included healthcare utilization rates, patient satisfaction, and health outcomes stratified by equity-relevant demographic factors. In digital health-focused frameworks, new metrics—such as user engagement with digital health platforms, digital health literacy levels, and stable access to secure digital tools—were proposed (18, 23, 33). These measures help quantify the extent to which digital health services reach and benefit underserved populations.

Enablers/Modifiers.

Policy reforms, community outreach, culturally tailored communication, funding mechanisms, training for both providers and patients, and user-centered technology design emerged as key enablers/modifiers. Digital health-specific frameworks recommended targeted interventions, such as offering multilingual digital health platforms, integrating digital health training into clinical curricula, subsidizing devices or Internet connectivity, and creating iterative feedback loops to improve the user experience continuously.

### 4.2 Applicability of frameworks to telehealth settings

From the quality assessment results presented in Additional File 4, we observe that eight frameworks received a score of 2 or higher in applicability, indicating that most frameworks are adaptable to telehealth settings to varying degrees. However, five frameworks scored only 1 point, highlighting notable limitations and gaps in their ability to comprehensively address the unique challenges of telehealth. Below, we explore the distinctions between digital health-specific frameworks and general frameworks.

#### 4.2.1 Digital health-specific frameworks

Frameworks explicitly designed for digital health often provide a more focused lens for assessing equity in telehealth. However, even among these, certain challenges persist (see Table 2).

#### 4.2.2 General frameworks

General health equity frameworks are broader in scope but reveal significant limitations when applied to telehealth contexts. Table 4 outlines common gaps, equity implications, and suggestions for improvement in these frameworks and telehealth strategies.

**Table 4:** Equity implications of limitations in current health equity frameworks applied to telehealth.

Gap/Limitation	Description	Equity implications	Suggestions for improvement
Limited focus on digital divide	Existing health equity frameworks often overlook the digital divide, which refers to disparities in access to information and communication technologies. This includes the availability and quality of Internet access, particularly in rural or underserved areas.	The digital divide leads to unequal access to telehealth services, potentially exacerbating health disparities for those without reliable Internet access or necessary digital tools.	For Telehealth Strategies: Develop targeted strategies to enhance digital infrastructure and provide subsidized access to technology in underserved regions.
			For Frameworks: Include digital connectivity as a key domain for assessing health equity in telehealth
Socioeconomic barriers	While Socioeconomic status is recognized as a driver of health equity, current frameworks may not fully address how telehealth interacts with these barriers. Telehealth can reduce some socioeconomic barriers, such as travel costs, but the costs of technology and Internet access can prevent vulnerable groups from benefiting fully.	Without addressing the costs and accessibility of technology and Internet services, telehealth may inadvertently widen the gap between different socioeconomic groups, limiting the potential benefits of digital healthcare for vulnerable populations.	For Telehealth Strategies: Incorporate considerations of Internet access costs and technological affordability into telehealth planning and subsidy programs.
			For Frameworks: Include socioeconomic status-specific measures for telehealth affordability and access as part of health equity evaluations.
Overlooking variations in digital literacy	Digital literacy, the ability to use digital technologies effectively, is often not addressed in traditional health equity frameworks. For telehealth to be effective, users need both access to technology and the skills to use it. Many individuals, particularly those with low health literacy, struggle with digital tools. This includes challenges related to age, cognitive abilities, and varying comfort levels with technology across cultural groups.	Low digital literacy can prevent individuals from effectively using telehealth services, leading to disparities in health outcomes. Different populations may face unique barriers that are not fully captured by current frameworks, necessitating more tailored approaches to digital health literacy.	For Telehealth Strategies: Include digital literacy training as part of telehealth services and create tailored educational programs for diverse populations.
			For Frameworks: Incorporate digital literacy as a critical component in evaluating telehealth equity.
Ignoring data privacy and security concerns	Data privacy and security are critical concerns in telehealth, but these are often insufficiently addressed in existing health equity frameworks. Concerns about how personal health information is collected, stored, and shared can deter people from using telehealth services. Even with encryption, platforms are vulnerable to data breaches, which could further erode trust in digital health solutions.	Insufficient focus on data privacy and security may reduce trust in telehealth services (e.g., among groups whose past experiences have led to poor trust in broader health systems), limiting their adoption and effectiveness. Addressing these concerns is essential for increasing user confidence and ensuring the broader acceptance of telehealth.	For Telehealth Strategies: Strengthen data protection measures, ensure compliance with stringent security standards, and enhance user education on data privacy.
			For Frameworks: Add data privacy and security as evaluative criteria to assess trust and adoption in telehealth systems.
Insufficient focus on health system integration	Telehealth needs to be integrated into the broader healthcare system to ensure quality and continuity of care. Current frameworks may not adequately address how to integrate telehealth with existing services, risking care fragmentation and reduced quality of care.	The lack of integration of telehealth into the broader healthcare system can result in fragmented care, which impacts underserved populations. Without seamless integration, these patients may experience gaps in care continuity, leading to poorer health outcomes and exacerbating existing health disparities. A more integrated approach is essential for achieving health equity.	For Telehealth Strategies: Develop guidelines to integrate telehealth services into the broader healthcare system to ensure continuity and quality of care.
			For Frameworks: Include integration with health systems as a dimension for assessing telehealth’s role in equity.

## 5 Discussion

This systematic review and narrative synthesis evaluated existing health equity frameworks to determine their applicability in telehealth contexts. We identified 13 unique frameworks across 17 included articles, spanning established models developed for broader healthcare settings and more recent frameworks designed for digital health. Six frameworks were explicitly created for digital health, telehealth, or eHealth contexts (Table 2), while the remaining seven were general health equity frameworks with potential applicability to telehealth (Additional File 3). Overall, the majority of the frameworks demonstrated moderate-to-high quality, with eight scoring ≥10/12 on our tailored appraisal (Additional File 4).

### 5.1 Contextualization within existing literature

Our findings align with prior reviews of health equity frameworks, which also report variation in how equity is conceptualized, with some frameworks emphasizing outcome disparities while others focus on access barriers (14, 25). Our review additionally highlights complexities introduced by digital health that earlier analyses did not address, including the role of digital determinants of health (18, 27, 32). We also observed that algorithmic bias was explicitly addressed in only one framework, underscoring the nascent nature of this area despite its recognized importance in the literature (27, 33). Finally, our appraisal identified Comprehensiveness as the weakest criterion, echoing long-standing concerns about the ability of frameworks to capture equity dimensions in a systematic way (11, 21).

### 5.2 Framework analysis and integration potential

Despite different origins and intended applications, substantial theoretical convergence is evident. As coded in our review (see Additional File 5), all frameworks acknowledge that health outcomes are shaped by social, economic, and environmental factors beyond medical care, and 11 of 13 explicitly incorporate cultural and linguistic considerations. Specific areas of convergence include access dimensions (seven frameworks) and policy or system-level interventions (six frameworks). Among digital health/eHealth frameworks, all six recognize digital determinants of health as equity drivers and extend the traditional social determinants to include technology-specific considerations, such as digital literacy and algorithmic bias. These counts and themes are detailed in Additional File 5.

This convergence suggests both theoretical maturity and opportunities for integration. The consistent appearance of core elements validates fundamental equity concepts and reveals redundancies across frameworks. Rather than proposing entirely new constructs, future development may benefit from explicit integration of existing elements into operational tools that can be applied consistently across telehealth services.

### 5.3 Applicability of frameworks to telehealth settings

Our appraisal of applicability (Section 4.2) showed that while the majority of the frameworks can be adapted to telehealth to some extent, important limitations remain. Digital health–specific frameworks provided clearer pathways by explicitly addressing digital determinants of health and emphasizing person-centered design, yet they often lacked operational metrics or required high resource commitments for equity assessment (27, 33). By contrast, general health equity frameworks offered a broad conceptual base but overlooked telehealth-specific domains such as digital literacy, privacy and security, and integration with health systems (see Tables 2, 4).

These omissions carry significant implications. A failure to account for the digital divide or the costs of connectivity may widen socioeconomic inequities. Insufficient attention to digital literacy and trust may reduce adoption among older adults, culturally diverse communities, or people with limited health literacy. Weak health system integration may fragment care and disproportionately affect underserved populations. Addressing these issues will require both adapting existing frameworks and developing tailored evaluation tools that explicitly incorporate digital access, literacy, privacy, and integration dimensions.

### 5.4 Implications for telehealth design and implementation (practice)

Our synthesis supports concrete design requirements for equitable telehealth delivery. Health services and platform vendors should implement: (1) digital inclusion by default (low-bandwidth options, device-agnostic access, and simplified onboarding); (2) supported use (digital navigation support, clear step-by-step prompts, and accessible alternatives when users cannot complete digital steps); and (3) culturally safe delivery (language support and culturally adapted workflows). Without these elements, telehealth may shift barriers rather than remove them. It can replace travel and time constraints with digital access, usability, and trust barriers, which may widen inequities for groups already experiencing disadvantage.

### 5.5 Implications for evaluation and measurement (minimum expectations)

Equity-focused telehealth evaluations should move beyond utilization alone and adopt a minimum set of stratified indicators across reach, use, experience/trust, and outcomes/continuity. At a minimum, evaluations should report: (a) reach (who was offered and able to access telehealth), (b) effective use (completion, drop-off points, and repeat use), (c) experience and trust (including perceived privacy/security and cultural alignment), and (d) care outcomes and continuity (including follow-up completion and integration with in-person care). Reporting should be stratified using standard equity characteristics (e.g., geography, socioeconomic position, ethnocultural/language groups, and age) to make inequities visible and enable targeted quality improvement. Without this, “equity-blind” evaluations risk overestimating benefit by averaging across groups with very different capacities to access and benefit from telehealth.

### 5.6 Implications for policy (what must be enabled)

Equity-oriented telehealth requires enabling policy settings that support digital connectivity and device access (particularly for rural/remote and low-income communities), workforce capacity for digital navigation and support, and standards for privacy, security, and interoperability that protect trust and prevent fragmentation of care. When these enabling conditions are absent, even well-designed services may systematically under-serve populations with constrained digital resources, lower digital literacy, or historical reasons for low institutional trust.

### 5.7 Operationalizing the integrated framework

Synthesizing these insights, we propose a three-level approach: (1) a Foundational level incorporating universal determinants (for example, socioeconomic status, geography, and culture) drawn from established frameworks such as WHO’s Social Determinants model and PROGRESS-Plus; (2) a Digital enhancement level layering technology-specific factors identified in digital health frameworks (for example, connectivity, device access, digital literacy, usability, and algorithmic fairness); and (3) an Implementation level providing practical guidance for assessment, intervention design, and evaluation, consistent with frameworks that emphasize monitoring, feedback, and continuous improvement. This multi-level integration addresses Comprehensiveness gaps identified in our appraisal while maintaining theoretical rigor.

**Figure 2:**
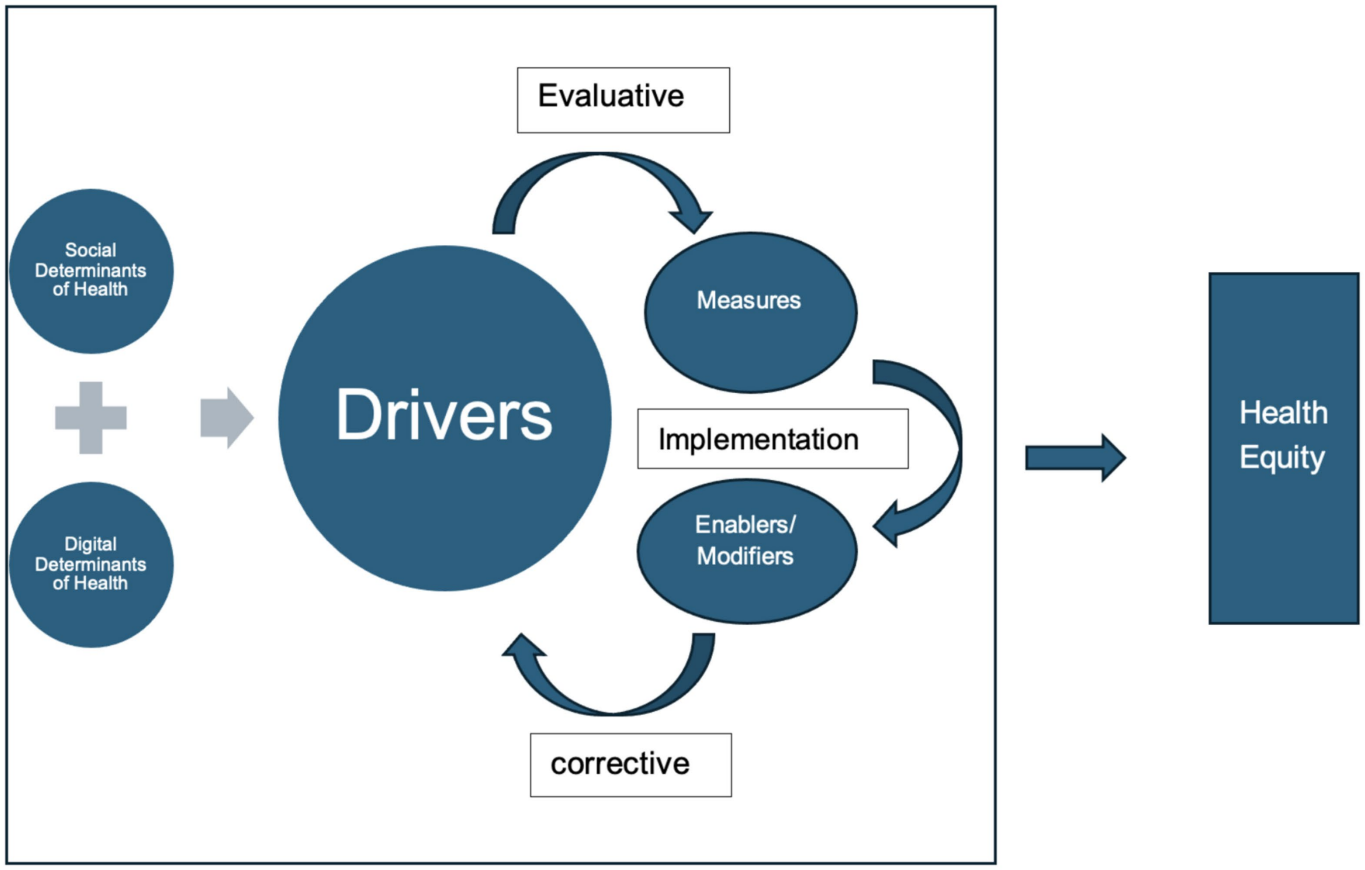
Proposed three-level integrated framework for assessing telehealth equity.

To translate the integrated framework into practice, we organized its elements into three functional components: drivers, measures, and enablers/modifiers. This structure provides a practical lens for assessing and addressing telehealth equity. Drivers include both foundational social determinants and emerging digital determinants, while measures extend beyond conventional utilization metrics to incorporate digital indicators such as telehealth adoption and sustained use. Enablers/modifiers encompass policy interventions, inclusive technology design, and digital literacy initiatives that shape implementation effectiveness. Together, these components support a systematic approach to embedding telehealth within broader strategies that address the social determinants of health. This operationalization is illustrated in Figure 2, which shows how drivers, measures, and enablers or modifiers interact through implementation and feedback loops to advance health equity. In doing so, the framework provides a practical basis for coordinated action and evaluation, and it sets out a clear program for further development and testing across diverse contexts.

### 5.8 Implications for future research

Future studies should validate and operationalize the integrated framework by testing it in real-world telehealth services across diverse settings, assessing the feasibility of routine equity reporting, and examining which implementation supports most effectively the reduction of observed inequities. This includes developing and evaluating practical reporting templates or checklists that services can adopt to standardize equity monitoring over time.

## 6 Limitations

A limitation is that data extraction was conducted by a single reviewer, and we did not calculate formal inter-rater reliability statistics. Screening, however, was performed independently by two reviewers with adjudication, and extracted information was cross-checked against the source articles during synthesis and table preparation to minimize transcription errors.

Only English-language publications were included in this review, which may have led to the exclusion of relevant studies published in other languages. Future research should consider including non-English studies to provide a more comprehensive perspective.

As this review focuses on conceptual frameworks rather than empirical studies, we developed a tailored quality appraisal tool to assess the Clarity, Theoretical Foundation, Comprehensiveness, and Applicability of each framework. While this approach allowed for a structured evaluation, the tool has not been externally validated, and its criteria may differ from those of established quality appraisal frameworks.

## 7 Conclusion

This systematic review examined existing health equity frameworks and assessed their applicability to telehealth. Many established frameworks, particularly those centered on social determinants, remain relevant but require deliberate adaptation to address challenges specific to telehealth, including connectivity and device access, digital literacy, usability and trust, and data and algorithmic fairness. Building on areas of convergence, we outline a three-part integration comprising foundational determinants, digital enhancements, and implementation guidance that clarifies how general and digital frameworks can be synthesized for telehealth evaluation and design. In doing so, we highlight the needs of underserved populations, especially rural and remote communities, and the importance of actionable measures to assess and mitigate digital health inequities. Priorities for future research include developing and validating an integrated telehealth equity framework; defining a core, stratified indicator set (access, use, experience, outcomes with PROGRESS Plus Stratifiers); conducting implementation studies with an equity focus that test framework utility in real services; pursuing longitudinal evaluations and evaluations across cultures; and advancing algorithmic equity through routine bias audits, representative data standards, and transparent reporting. Addressing these priorities will help ensure digital health functions as a leveler rather than a divider, providing researchers, providers, and policymakers with practical tools to design, implement, and evaluate equitable telehealth.

## Data Availability

The original contributions presented in the study are included in the article/Supplementary material, further inquiries can be directed to the corresponding author.
