# Supplementary material for: Evaluation of health equity frameworks in telehealth and digital health: a systematic review and narrative synthesis
Source: Front Public Health. 2026 Jan 6;13:1690117. doi: 10.3389/fpubh.2025.1690117 (PMC12815789; doi:10.3389/fpubh.2025.1690117)
Supplement: Supplementary file 1 [file Table_1.DOCX]

**Additional file 1** Information sources and search log

| **Source** | **Platform / interface** | **Run(s)** | **Search date (month/year)** | **Records retrieved (combined across runs)** | **Notes** |
| --- | --- | --- | --- | --- | --- |
| MEDLINE | Ovid | Initial + update | 2 Jul 2024; 11 Nov 2024 | 332 | Strategy in Additional File 2 |
| CINAHL | EBSCOhost | Initial + update | 2 Jul 2024; 11 Nov 2024 | 232 | Strategy in Additional File 2 |
| Scopus | Elsevier | Initial + update | 2 Jul 2024; 11 Nov 2024 | 143 | Strategy in Additional File 2 |
| Citation searching | Scopus / manual | Initial + update | NA | 8 | Yield added to full-text screening |
