# Supplementary material for: Evaluation of health equity frameworks in telehealth and digital health: a systematic review and narrative synthesis
Source: Front Public Health. 2026 Jan 6;13:1690117. doi: 10.3389/fpubh.2025.1690117 (PMC12815789; doi:10.3389/fpubh.2025.1690117)
Supplement: Supplementary file 2 [file Table_2.DOCX]

**Additional file 2.**

Part A: Search strategies for different databases

| **Database** | **Search Fields** | **Search String(s)** | **Search Run Dates** | **Limits** |
| --- | --- | --- | --- | --- |
| SCOPUS | Article title, Abstract, Keywords | (telehealth or telemedicine or "e-health" or ehealth or "digital health" or "video consult*" or "remote consult*")  AND  ((framework* OR model* OR assess* OR evaluat*) W/5 (equity OR equal* OR inequalit* OR disparit*)) | 2 July 2024 (initial); 11 November 2024 (update) | English only |
| MEDLINE | Keyword | 1. (telehealth.mp. or Telemedicine)  2. (telemedicine.mp.)  3. (e-health.mp.)  4. (ehealth.mp.)  5. (digital health.mp. or Digital Health/ or Mobile Applications)  6. (Remote Consultation/ or Remote Consult*.mp.)  7. (video consult*.mp.)  8. (virtual care.mp.)  9. 1 or 2 or 3 or 4 or 5 or 6 or 7 or 8  10. ((equity or equal* or inequalit* or disparit*) adj5 (framework* or model* or assess* or evaluat*)).mp.  11. 9 AND 10 | 2 July 2024 (initial); 11 November 2024 (update) | English only |
| CINAHL | MH/TI/AB/Keywords | S1 (MH "Telehealth+") OR "telehealth"  S2 (MH "Telemedicine+") OR "telemedicine"  S3 TI (“e-health" OR ehealth OR "digital health”) OR AB (“e-health" OR ehealth OR "digital health”)  S4 (MH "Remote Consultation") OR "video consultation" OR (MH "Telephone Consultation (Iowa NIC)")  S5 “virtual care"  S6 "telecare"  S7 S1 OR S2 OR S3 OR S4 OR S5 Or S6  S8 (framework* OR model* OR assess* OR evaluat*) N5 (equity OR equal* OR inequalit* OR disparit*)  S9 S7 AND S8 | 2 July 2024 (initial); 11 November 2024 (update) | English only |

Part B: Full-text exclusion log (n = 10)

| **Full-text record** | **Reason for exclusion** |
| --- | --- |
| Baquero, B., Gonzalez, C., Ramirez, M., Chavez Santos, E., & Ornelas, I. J. (2020). Understanding and Addressing Latinx COVID-19 Disparities in Washington State. Health Education & Behavior, 47(6), 845–849. https://doi.org/10.1177/1090198120963099 | Lack of health equity discussion |
| Bitomsky, L., Pfitzer, E. C., Nißen, M., & Kowatsch, T. (2024). Advancing health equity and the role of digital health technologies: a scoping review protocol. BMJ Open, 14(10), e082336. https://doi.org/10.1136/bmjopen-2023-082336 | Lack of health equity discussion (protocol only) |
| Blanc, J., Hahn, K., Oliveira, B., Phillips, R., Duthely, L. M., Francois, L., Carrasco, M., Moore, J., Sternberg, C. A., Jean-Louis, G., & Seixas, A. A. (2023). Bringing Health Care Equity to Diverse and Underserved Populations in Sleep Medicine and Research Through a Digital Health Equity Framework. Sleep Medicine Clinics, 18(3), 255–267. https://doi.org/10.1016/j.jsmc.2023.05.009 | Focuses primarily on clinical/technical outcomes (not framework-focused for this review) |
| Hartwell, M., Lin, V., Gatewood, A., Sajjadi, N. B., Garrett, M., Reddy, A. K., Greiner, B., & Price, J. (2022). Health disparities, COVID-19, and maternal and childbirth outcomes: a meta-epidemiological study of equity reporting in systematic reviews. Journal of Maternal-Fetal & Neonatal Medicine, 35(25), 9622–9630. https://doi.org/10.1080/14767058.2022.2049750 | Focuses primarily on clinical/technical outcomes |
| Steinman, L., Chavez Santos, E., Chadwick, K., Mayotte, C., Johnson, S. S., Kohn, M., Kelley, J., Denison, P., Montes, C., Spencer-Brown, L., & Lorig, K. (2024). Remote Evidence-Based Health Promotion Programs During COVID: A National Evaluation of Reach and Implementation for Older Adult Health Equity. Health Promotion Practice, 25(3), 475–491. https://doi.org/10.1177/15248399231175843 | Focuses primarily on clinical/technical outcomes |
| Ochtli, C., Gibran, N., Mandell, S., Pham, T., & Stewart, B. (2019). A Health Equity Framework for Telemedicine at a Regional Burn Center. Journal of Burn Care & Research, 40(Supplement_1), S14–S15. https://doi.org/10.1093/jbcr/irz013.021 | Focuses primarily on clinical/technical outcomes |
| Partridge, S. R., Knight, A., Todd, A., McGill, B., Wardak, S., Alston, L., Livingstone, K. M., Singleton, A., Thornton, L., Jia, S., Redfern, J., & Raeside, R. (2024). Addressing disparities: A systematic review of digital health equity for adolescent obesity prevention and management interventions. Obesity Reviews, 25(12), e13821. https://doi.org/10.1111/obr.13821 | Focuses primarily on clinical/technical outcomes |
| Kaihlanen, A. M., Virtanen, L., Buchert, U., Safarov, N., Valkonen, P., Hietapakka, L., Hörhammer, I., Kujala, S., Kouvonen, A., & Heponiemi, T. (2022). Towards digital health equity - a qualitative study of the challenges experienced by vulnerable groups in using digital health services in the COVID-19 era. BMC Health Services Research, 22(1), 188. https://doi.org/10.1186/s12913-022-07584-4 | Existing framework application only (no new/unique framework) |
| Nair, U. S., Kue, J., Athilingam, P., Rodríguez, C. S., & Menon, U. (2023). Application of the ConNECT Framework to achieve digital health equity. Nursing Outlook, 71(4), 101991. https://doi.org/10.1016/j.outlook.2023.101991 | Existing framework application only (no new/unique framework) |
| Raza, M. M., Venkatesh, K. P., & Kvedar, J. C. (2023). Promoting racial equity in digital health: applying a cross-disciplinary equity framework. NPJ Digital Medicine, 6(1), 3. https://doi.org/10.1038/s41746-023-00747-5 | Non-academic literature (Editorial) |
