# Supplementary material for: Evaluation of health equity frameworks in telehealth and digital health: a systematic review and narrative synthesis
Source: Front Public Health. 2026 Jan 6;13:1690117. doi: 10.3389/fpubh.2025.1690117 (PMC12815789; doi:10.3389/fpubh.2025.1690117)
Supplement: Supplementary file 3 [file Table_3.DOCX]

**Additional file 3.** Summary of Health Equity Frameworks, with the main purpose and key elements as described by the framework authors.

| **Reference(s)** | **Framework/Model Name** | **Main Purpose** | **Key Elements** |
| --- | --- | --- | --- |
| Aday & Andersen, 1974 [27](#_ENREF_27) | Aday and Andersen Model | This framework is primarily used to study the determinants of healthcare service utilization, focusing on why individuals use healthcare services and how the opportunities for different populations to access healthcare vary. | (1) **Health policy** (financing, education, manpower, health care reorganization programs)  (2) **Characteristics of the health care delivery system**:  - Resources (volume and the distribution)  - Organizations (entry, structure)  (3) **Characteristics of the population at risk**:  - Predisposing (age, sex, race, religion, and values concerning health and illness)  - Enabling (income, insurance coverage,  rural-urban character, region)  - Need (perceived, evaluated)  (4) **Utilization of health care services** (type, sites, purpose, time interval)  (5) **Consumer satisfaction** (convenience, costs, coordination, courtesy, information, quality) |
| Kepper et al., 2024 [28](#_ENREF_28) | A Model for Advancing Digital Health Access to Foster Health Equity | This model aims to advance health equity by identifying and addressing multilevel factors that drive inequities in access to and utilization of digital health tools. | (1) **Multilevel Access Dimensions:**  Adapted from Levesque et al.’s framework, the model focuses on approachability, acceptability, availability, affordability, and appropriateness of digital health tools to address access barriers comprehensively.  (2) **Design, Delivery, Dissemination, and Sustainability:**  -Design: User-centered, participatory approaches to ensure inclusivity and cultural relevance.  -Delivery: Incorporating workflows, training, and technical support for seamless integration.  -Dissemination: Targeted outreach to raise awareness among underserved populations.  -Sustainability: Developing funding models and infrastructure to support long-term accessibility.  (3) **Evaluation and Ethics:**  -Emphasizes ongoing evaluation of access metrics and ethical principles to ensure tools are effective, inclusive, and aligned with the needs of diverse communities. |
| World Health Organisation, 2010 [22](#_ENREF_22) | Conceptual Framework for Action on the Social Determinants of Health | This framework offers a comprehensive approach to understanding and addressing the social determinants of health (SDoH) and their impact on health inequalities. The framework aims to elucidate the fundamental processes that lead to health inequalities through a “causal” perspective. As an “action-oriented” framework, it is designed to assist policymakers in identifying where to intervene in these fundamental processes to most effectively combat health inequalities. | (1) **Socio-economic and political context**, which are the backgrounds that generate social stratification and class divisions:  -Governance: Policies and governance structures that impact health outcomes.  -Macroeconomic Policies: Economic policies affecting income distribution, employment, and social security.  -Social Policies: Education, health, labour market, and housing policies.  -Public Policies: Including both social and economic policies.  -Culture and Societal Values: The broader social norms and cultural values that influence social determinants of health.  -Epidemiological Conditions: particularly in the case of major epidemics such as HIV/AIDS, which exert a powerful influence on social structures and must be factored into global and national policy setting.  (2) **Structural determinants of health inequalities**, which shape the conditions of inequality:  -Socioeconomic Position: The social and economic status of individuals, determined by factors such as income, education, occupation, gender, race/ethnicity, and social class.  -Social Stratification: The division of society into hierarchical groups, affecting access to resources and opportunities.  (3) **Intermediary determinants of health**, formed by structural determinants—housing, physical work environment, social support, stress, nutrition and physical activity—to shape individual health outcomes:  -Material Circumstances: Living and working conditions, food availability, housing quality, and neighbourhood characteristics.  -Behaviours and Biological Factors: Health-related behaviours (smoking, diet, physical activity) and biological/genetic factors.  -Psychosocial Factors: Stress, social support, and coping mechanisms.  -Health System: Accessibility, affordability, and quality of health care services. |
| Levesque et al., 2013 [18](#_ENREF_18)  Pullyblank et al., 2023 [21](#_ENREF_21) | Conceptual Framework of Access to Health Care | This framework emphasizes the complexities of access and identifies factors that can enhance or hinder access to health services. These dimensions originate from both the supply side (such as healthcare systems and organizations) and the demand side (such as individuals, families, and communities), involving different processes that facilitate accessibility (such as healthcare needs, healthcare-seeking behaviour, and healthcare coverage). | (1) **Approachability** (Perceived Ability): Recognizes whether health services are known to exist, can be reached, and are open to all.  (2) **Acceptability** (Seeking Ability): Relates to the match between how responsive health services are to the cultural and social expectations of individual users.  (3) **Availability and Accommodation** (Reaching Ability): Ensures that services are available at appropriate times and supported by sufficient resources and staff.  (4) **Affordability** (Paying Ability): Considers the economic capacity of individuals to spend resources without causing hardship.  (5) **Appropriateness** (Engaging Ability): Measures the fit between health services and client needs, ensuring that services are provided in a respectful and coordinated manner that is relevant to the health needs. |
| Crawford & Serhal, 2020 [30](#_ENREF_30) | Digital Health Equity Framework (DHEF) | This framework aims to address health equity in the design, implementation, and evaluation of digital health solutions. It focuses on identifying and mitigating digital determinants of health that may reinforce or exacerbate existing health inequities. It also integrates digital health equity considerations into health systems and provider practices to ensure inclusive access and equitable health outcomes. | (1) **Social Stratification:**  -Incorporates the hierarchical allocation of resources and power based on intersectional factors like race, income, age, gender, and geography.  -Links these factors to a person’s health risks and material circumstances.  (2) **Digital Determinants of Health**:  -Includes access to technology, digital health literacy, and psychosocial factors influencing the usage of digital health services.  -Considers the impact of digital health access on intermediate health outcomes, such as beliefs, behaviours, and preexisting conditions.  (3) **Systemic and Institutional Context:**  -Highlights the role of institutions, policies, and health systems in shaping digital health outcomes.  -Encourages the development of equitable health strategies and culturally safe environments.  (4) **Ecological Approach:**  -Examines the interplay between individual behaviours, social circumstances, and broader systemic factors.  -Advocates for integrating health equity into the digital health ecosystem from development to implementation.  (5) **Measurement and Evaluation:**  -Emphasizes the need for systematic measurement of digital health equity outcomes.  -Calls for the collection and analysis of health equity data to refine the framework and guide policy changes.  (6) **Person-Centered Care:**  -Ensures digital health solutions are adaptable to diverse needs and empower patient autonomy.  -Encourages inclusion of vulnerable populations in the design and leadership of digital health innovations. |
| Groom et al., 2024 [29](#_ENREF_29) | Digital Health Equity-focused Implementation Research Model (DH-EquIR) | To guide the planning, design, implementation, and evaluation of digital health interventions with a focus on promoting health equity. It aims to address systemic inequities by integrating equity-focused strategies across all stages of digital health implementation. | (1) **Population Health Status and Determinants of Health:**  -Combines social determinants of health (SDOH) and digital determinants of health (DDOH) to assess population needs and inequities.  (2) **Planning the Program:**  -Identifies disadvantaged groups.  -Quantifies current health inequities.  -Develops equity-sensitive recommendations tailored to specific needs.  (3) **Designing the Program:**  -Identifies key actors, including community members and facilitators.  -Addresses barriers to equity-focused recommendations.  -Incorporates culturally and contextually relevant design principles.  (4) **Implementing the Program:**  -Designs equity-focused communication strategies.  -Defines resources and incentives for implementation.  -Develops strategies to overcome identified barriers.  (5) **Equity-Focused Implementation Outcomes:**  -Measures sustainability, fidelity, and adoption rates.  -Tracks the impact on targeted populations and evaluates success using defined equity metrics. |
| Antonio & Petrovskaya, 2019 [33](#_ENREF_33) | eHealth Equity Framework (eHEF) | The Framework aims to integrate health equity considerations throughout the lifecycle of health information technologies (HITs), such as electronic health records and patient portals. It is designed to address inequities inadvertently perpetuated by eHealth solutions, ensuring that health equity remains a core goal in designing, implementing, and evaluating digital health interventions. | (1) **Socio-Techno-Economic-Political Context**:  -Incorporates factors like governance, policy, cultural values, and existing technologies that influence health equity.  -Highlights how existing technological infrastructure can both enable and constrain equitable access.  (2) **Patients’ Social Position and Characteristics:**  -Accounts for education, occupation, income, gender, age, ethnicity, race, and geographic location as socially mediated determinants.  -Emphasizes intersectionality to address the complex ways social determinants affect patient outcomes.  (3) **Intermediary Determinants of Health:**  -Includes technology and healthcare access, material circumstances, and social capital.  -Recognizes the importance of multiple forms of literacy (health, digital, and media literacy) in effective technology use.  (4) **Technology Integration:**  -Incorporates technology into every stage of the framework, from pre-existing infrastructure to implementation and outcomes.  -Acknowledges that technology can both exacerbate inequities and serve as a tool to address them.  (5) **Life Course Perspective:**  -Reflects the impact of socio-economic and political contexts throughout the lifecycle of health information technologies.  (6) **Feedback Loops and Non-Linear Processes:**  -Encourages an iterative approach to understanding and addressing inequities through continuous evaluation and adaptation. |
| Richardson et al., 2022 [32](#_ENREF_32) | Framework for Digital Health Equity | The framework provides a detailed explanation of the key Digital Determinants of Health (DDoH) to support the work of industry professionals, healthcare system operators, and academic researchers in the creation of digital health tools. The DDoH are studied at the individual, interpersonal, community, and societal levels, discussing the root causes and the importance of a multi-level approach. | (1) At the **individual level**, the determinants include digital literacy, digital self-efficacy, technology acquisition, and attitudes towards technology use.  (2) At the **interpersonal level**, the determinants encompass hidden technology biases, interdependencies, and the relationships between patients, technology professionals, and clinicians.  (3) At the **community level**, the determinants cover community infrastructure, healthcare infrastructure, community technology standards, and community partnerships.  (4) At the **societal level**, the determinants involve technology policies, data and design standards, social norms and ideologies, and algorithmic biases. |
| Woodward et al., 2019 [24](#_ENREF_24)  Woodward et al., 2021 [25](#_ENREF_25)  Norman et al., 2024 [19](#_ENREF_19) | Health Equity Implementation Framework (HEIF) | The HEIF primarily focuses on integrating health equity principles into specific health programs and policies. Its purpose is to identify the determinants of health equity so that interventions and implementation strategies can be adjusted or tailored to promote health equity. This framework aims to ensure that health interventions effectively reduce health disparities, especially among populations with lower socio-economic status or limited resources. | (1) **Culturally related factors**, such as mistrust in healthcare, demographic biases, or prejudices of recipients;(2) **Clinical encounters** or interactions between patients and providers; (3) **Social contexts**, including material infrastructure, economics, and the social and political forces at play. |
| Dover & Belon, 2019 [17](#_ENREF_17) | Health Equity Measurement Framework (HEMF) | The HEMF provides a comprehensive view of numerous social determinants of health (SDoH) and the drivers of healthcare utilization, along with guiding quantitative analysis for public health monitoring and policy making to address these disparities. | This framework encompasses socio-economic, cultural, and political contexts; health policy background; social stratification and status; material and social environments; environmental and biological factors; health-related behaviours and beliefs; stress; care quality; and healthcare utilization. |
| O’Neill et al., 2014 [20](#_ENREF_20)  Woolley et al., 2023 [26](#_ENREF_26) | PROGRESS/PROGRESS-PLUS | PROGRESS-Plus is a framework used to identify and analyse health inequalities across various dimensions. It is designed to enhance understanding and reporting of equity issues in health research and interventions. | (1) **Place of residence**: Urban/rural location, region, country.  (2) **Race/ethnicity/culture/language**: Different racial or ethnic groups, cultural practices, and language barriers.  (3) **Occupation**: Type of employment, working conditions, and employment status.  (4) **Gender/sex**: Differences based on gender and biological sex.  (5) **Religion**: Religious beliefs and practices influencing health.  (6) **Education**: Level of education attained and its impact on health literacy and behaviours.  (7) **Socioeconomic status**: Income level, wealth, and social class.  (8) **Social capital**: Social networks, community support, and social cohesion.  The “PLUS” component captures additional factors that may be relevant in specific contexts:  (1) **Personal characteristics**: Age, disability, sexual orientation.  (2) **Features of relationships**: Family structure, marital status.  (3) **Time-related factors**: Life cycle, duration of exposure to certain conditions. |
| Szymczak et al., 2023 [23](#_ENREF_23) | Process Model of Healthcare Access, Quality and Equity | This model illustrates the impact of telehealth on patient, population, and system outcomes, and is intended to be used to broadly guide research, practice, and policy concerning the equity impacts of healthcare service innovations. | (1) **Patient Care Seeking**, including:  - Care Seeking: Identifying and perceiving health needs.  - Care reaching: The ability to access healthcare services, which can be affected by geographic, financial, and other barriers.  - Care Encounters: Interactions with healthcare providers and receiving care.  - Health Outcomes: Results of medical interventions over a period.  (2) **Care Delivery Systems**, which includes:  - Representing System People: Healthcare professionals and support staff.  - Processes: The procedures and protocols that govern how care is delivered, including the use of tools and technology.  - Environment: The physical and organizational setting in which care is provided.  (3) **Systemic Commitment to Health Equity**:  -Anti-Oppression Frameworks: Initiatives and policies designed to address and dismantle oppressive practices within healthcare systems. This includes efforts to repair, remediate, restructure, and remove barriers to equitable care.  -Feedback Loops: Mechanisms that allow the system to learn from the outcomes and adapt processes to improve both access and quality of care. These loops are critical for making ongoing adjustments that enhance system performance and patient outcomes.  (4) **Outcomes**:  -System Outcomes: Metrics such as quality, safety, efficiency, cost-effectiveness, healthcare worker morale, and trustworthiness of the healthcare system.  -Patient Outcomes: Direct impacts on patients, including health outcomes, satisfaction with care, costs, and trust in the system.  -Population Outcomes: Broader impacts on health equity, such as access and outcome disparities across different demographic groups (e.g., race, ethnicity, language, payer). |
| Foley et al., 2021 [31](#_ENREF_31) | Suggested Pathways of Access, Use and Benefit from Digital Health Services | This framework was developed to explore and enhance equity in access to, use of, and benefits derived from population-oriented digital health services. | (1) **Access:**  -Availability and affordability of technology.  -Sociodemographic barriers like age, socioeconomic status, and education.  -Technological literacy and infrastructure.  (2) **Use:**  -Trust in digital health services.  -Confidence in navigating and using digital tools effectively.  -Integration of digital services with face-to-face healthcare.  (3) **Benefit:**  -Improved health literacy and self-efficacy.  -Complementarity between digital and face-to-face services.  -Empowerment in health-related decision-making.  (4) **Social Determinants of Health:**  -Incorporates the broader social context affecting digital health equity, including geographic location, cultural background, and economic disparities.  (5) **Trust and Literacy:**  -Highlights the foundational role of trust in digital tools and services.  -eHealth literacy as a critical mediator for effective use and benefit. |
