# Supplementary material for: Evaluation of health equity frameworks in telehealth and digital health: a systematic review and narrative synthesis
Source: Front Public Health. 2026 Jan 6;13:1690117. doi: 10.3389/fpubh.2025.1690117 (PMC12815789; doi:10.3389/fpubh.2025.1690117)
Supplement: Supplementary file 4 [file Table_4.DOCX]

**Additional file 4**

Part A: the rubric table (Criterion × 0-3 anchors)

| **Criterion** | **0 (Not met)** | **1 (Partially met)** | **2 (Mostly met)** | **3 (Fully met)** |
| --- | --- | --- | --- | --- |
| Clarity of purpose | No clear aim/target context/intended users stated. | Purpose stated but vague; limited specification of population/setting or intended use. | Purpose and intended use described with minor ambiguity. | Explicit purpose, target context/population, and intended users/use-cases clearly stated. |
| Theoretical basis | No theory/conceptual foundations referenced. | Implicit or minimal theoretical grounding; cites concepts without explaining how they inform the framework | Relevant theory/evidence identified with partial integration. | Clear theoretical grounding and explicit explanation of how it informed development. |
| Comprehensiveness (telehealth-relevant equity domains) | Very limited domains; major determinants absent. | Some core domains included but multiple important domains omitted. | Most key domains covered with minor omissions/limited depth. | Comprehensive coverage including social and digital determinants relevant to telehealth/digital health. |
| Applicability to telehealth | No guidance for application/assessment in telehealth. | Mentions relevance but offers limited practical guidance. | Some practical guidance but limited operational detail. | Clear actionable guidance for telehealth application (steps, measurable constructs/indicators, implementation considerations). |

Part B: the Blank Scoring Template

| **Criterion** | **Score (0–3)** | **Justification (brief, evidence-based)** |
| --- | --- | --- |
| Clarity of purpose |  |  |
| Theoretical basis |  |  |
| Comprehensiveness (telehealth-relevant equity domains) |  |  |
| Applicability to telehealth |  |  |

Scoring: 0–3 each, total 0-12; High 10-12 / Moderate 7-9 / Fair 4-6 / Low 0-3.

Part C: Quality and applicability assessment of included frameworks

| **Authors/Framework name** | **Clarity of Purpose** | **Theoretical Basis** | **Comprehensiveness** | **Applicability** | **Total Score (0–12)** |
| --- | --- | --- | --- | --- | --- |
| Foley et al., 2021 | 1 | 3 | 1 | 2 | 7 |
| Pullyblank et al., 2023 Access to health care model | 3 | 2 | 3 | 3 | 11 |
| Antonio & Petrovskaya, 2019 **eHealth Equity Conceptual Framework** | 3 | 3 | 2 | 1 | 9 |
| Richardson et al., 2022 **Framework for digital health equity** | 3 | 2 | 2 | 3 | 10 |
| Szymczak et al., 2023 Process model of healthcare access, quality and equity | 2 | 3 | 3 | 3 | 11 |
| WHO, 2010 A Conceptual Framework for Action on the Social Determinants of Health | 2 | 3 | 2 | 1 | 8 |
| Woodward et al., 2019 Health Equity Implementation Framework | 3 | 3 | 3 | 2 | 11 |
| Woodward et al., 2021 Health Equity Implementation Framework | 3 | 3 | 2 | 2 | 10 |
| Woolley et al., 2023 PROGRESS PLUS | 2 | 2 | 1 | 2 | 7 |
| Aday & Andersen, 1974 | 3 | 3 | 2 | 1 | 9 |
| Dover & Belon, 2019 Health Equity Measurement Framework | 3 | 3 | 1 | 1 | 8 |
| Crawford & Serhal, 2020 **The Digital Health Equity Framework** | 1 | 2 | 1 | 3 | 7 |
| Kepper et al., 2024 **A model for advancing digital health access to foster health equity** | 2 | 3 | 2 | 3 | 10 |
| Groom et al., 2024 **Digital Health Equity-focused Implementation Research Model** | 3 | 3 | 2 | 3 | 11 |
| Levesque et al., 2013 A conceptual framework of access to health care | 3 | 3 | 2 | 2 | 10 |
| Norman et al., 2023 Health Equity Implementation Framework | 3 | 1 | 2 | 3 | 9 |
| O'Neill et al., 2014 PROGRESS | 3 | 1 | 1 | 1 | 6 |
| Note: Frameworks related to digital health or eHealth are highlighted in bold. | | | | | |
