# Supplementary material for: Evaluation of health equity frameworks in telehealth and digital health: a systematic review and narrative synthesis
Source: Front Public Health. 2026 Jan 6;13:1690117. doi: 10.3389/fpubh.2025.1690117 (PMC12815789; doi:10.3389/fpubh.2025.1690117)
Supplement: Supplementary file 5 [file Table_5.DOCX]

**Additional file 5** Common Factors and Key Differences Across Health Equity Frameworks

| **Category** | **Factor** | **Description and the Impact of Factors on Health Equity** | **Frameworks/Models with the factor** |
| --- | --- | --- | --- |
| **Common Factors** | Social Determinants of Health (SDoH) | Emphasize that broad social, economic, cultural, and political factors fundamentally shape health opportunities and outcomes. Addressing SDoH can reduce structural disparities and improve equitable health outcomes. | All frameworks |
|  | Cultural and Linguistic Appropriateness/Cultural Competence | Stresses matching healthcare services with patients’ cultural, linguistic, and social contexts to build trust and engagement. | 1. Conceptual Framework for Action on the Social Determinants of Health 2. Framework for Digital Health Equity 3. HEIF 4. HEMF 5. PROGRESS-PLUS 6. Process Model of Healthcare Access, Quality and Equity 7. DHEF 8. DH-EquIR 9. eHEF 10. A Model for Advancing Digital Health Access to Foster Health Equity 11. Suggested Pathways of Access, Use and Benefit from Digital Health Services |
|  | Access Dimensions | Includes availability, affordability, acceptability, approachability, and appropriateness of services. | 1. Aday and Andersen Model 2. A Model for Advancing Digital Health Access to Foster Health Equity 3. Conceptual Framework for Action on the Social Determinants of Health 4. Conceptual Framework of Access to Health Care 5. DHEF 6. Process Model of Healthcare Access, Quality and Equity 7. Suggested Pathways of Access, Use and Benefit from Digital Health Services |
|  | DDoH (in Digital/ehealth Contexts) | Disparities in access to healthcare arise due to unequal access to digital technologies, such as devices, reliable internet connections, and the skills needed to use them effectively. Core factor leading to disparities in access to healthcare. | 1. DHEF 2. eHEF 3. Framework for Digital Health Equity 4. DH-EquIR 5. A Model for Advancing Digital Health Access to Foster Health Equity 6. Suggested Pathways of Access, Use and Benefit from Digital Health Services |
|  | Policy and Governance Interventions | Identifies the influence of policies, regulations, and resource allocation at systemic levels on health equity and telehealth access. | 1. Aday and Andersen Model 2. Conceptual Framework for Action on the Social Determinants of Health 3. DHEF 4. eHEF 5. HEMF 6. Process Model of Healthcare Access, Quality and Equity |
|  | Iterative Feedback and Continuous Evaluation | Emphasizes ongoing monitoring, feedback loops, and adaptive approaches to continually address and improve health equity outcomes. | 1. Aday and Andersen Model 2. Process Model of Healthcare Access, Quality and Equity 3. eHEF 4. A Model for Advancing Digital Health Access to Foster Health Equity 5. DHEF 6. DH-EquIR |
| **Differences** | Algorithmic Bias & Advanced Digital Determinants | Highlights specific concerns about algorithmic fairness and data biases in digital tools. Addressing these factors could lead to more equitable data-driven interventions. | Framework for Digital Health Equity |
|  | Life Course Perspective | Considers health equity across the lifespan. Measures how health equity evolves over time, influenced by age and life stages. | 1. eHEF 2. PROGRESS-PLUS |
|  | Person-Centered Design | Ensuring inclusivity and accessibility through user-centered approaches. | 1. DHEF 2. DH-EquIR 3. A Model for Advancing Digital Health Access to Foster Health Equity |
|  | Precise Quantitative Digital Equity Metrics | Focuses on specialized measures for digital health equity. These metrics can improve the precision of interventions and allows tracking of progress toward more equitable digital health outcomes. | 1. DHEF 2. DH-EquIR 3. A Model for Advancing Digital Health Access to Foster Health Equity 4. Process Model of Healthcare Access, Quality and Equity |
